# Characterization and Chondroprotective Effects of Extracellular Vesicles From Plasma- and Serum-Based Autologous Blood-Derived Products for Osteoarthritis Therapy

**DOI:** 10.3389/fbioe.2020.584050

**Published:** 2020-09-25

**Authors:** Alexander Otahal, Karina Kramer, Olga Kuten-Pella, René Weiss, Christoph Stotter, Zsombor Lacza, Viktoria Weber, Stefan Nehrer, Andrea De Luna

**Affiliations:** ^1^Center for Regenerative Medicine, Department for Health Sciences, Medicine and Research, Danube University Krems, Krems an der Donau, Austria; ^2^OrthoSera GmbH, Krems an der Donau, Austria; ^3^Center for Biomedical Technology, Department for Biomedical Research, Danube University Krems, Krems an der Donau, Austria; ^4^Deptartment Sports Physiology, University of Physical Education, Budapest, Hungary

**Keywords:** autologous blood product, platelet rich plasma, hyperacute serum, extracellular vesicles, exosomes, gene expression, osteoarthritis, chondrocytes

## Abstract

Autologous blood products gain increasing interest in the field of regenerative medicine as well as in orthopedics, aesthetic surgery, and cosmetics. Currently, citrate-anticoagulated platelet-rich plasma (CPRP) preparations are often applied in osteoarthritis (OA), but more physiological and cell-free alternatives such as hyperacute serum (hypACT) are under development. Besides growth factors, blood products also bring along extracellular vesicles (EVs) packed with signal molecules, which open up a new level of complexity at evaluating the functional spectrum of blood products. Large proportions of EVs originated from platelets in CPRP and hypACT, whereas very low erythrocyte and monocyte-derived EVs were detected via flow cytometry. EV treatment of chondrocytes enhanced the expression of anabolic markers type II collagen, SRY-box transcription factor 9 (SOX9), and aggrecan compared to full blood products, but also the catabolic marker and tissue remodeling factor matrix metalloproteinase 3, whereas hypACT EVs prevented type I collagen expression. CPRP blood product increased SOX9 protein expression, in contrast to hypACT blood product. However, hypACT EVs induced SOX9 protein expression while preventing interleukin-6 secretion. The results indicate that blood EVs are sufficient to induce chondrogenic gene expression changes in OA chondrocytes, while preventing proinflammatory cytokine release compared to full blood product. This highlights the potential of autologous blood-derived EVs as regulators of cartilage extracellular matrix metabolism and inflammation, as well as candidates for new cell-free therapeutic approaches for OA.

## Introduction

Blood-derived products comprise plasma- or serum-based whole blood derivatives. They have been applied to diseased joints of osteoarthritis (OA) patients via intra-articular injection for over a decade. The efficacy of blood products especially with focus on knee OA has been investigated in several clinical trials and reviewed extensively. In general, blood products are well tolerated and provide reduction of pain and inflammation, as well as increased range of motion (Fotouhi et al., 2018; Tassara et al., 2018; Zarringam et al., 2018; Gato-Calvo et al., 2019). Autologous blood products are subfractions of whole blood drawn from a patient and are produced directly in the operation theater or for ambulatory treatment prior to application. In the context of orthopedics, platelet-rich plasma (PRP) is most commonly used and is characterized by a platelet concentration that exceeds the physiological concentration. To take effect, PRP has to be activated to induce platelet degranulation, which releases a number of growth factors such as platelet-derived growth factor, transforming growth factor β (TGF-β), insulin-like growth factor, or epidermal growth factor (Marx, 2004; Leitner et al., 2006; Qian et al., 2017). Upon intra-articular injection of non-activated PRP into a defect, PRP can be activated by contact with local tissue factor (Vanschoonbeek et al., 2004). Commercial preparation procedures employ different types of anticoagulants including citrate, EDTA, and heparin, as well as different centrifugation parameters, resulting in products with distinct residual leukocyte and platelet concentration profiles. At least 21 different PRP production devices are currently available and are applied without standardization (Gato-Calvo et al., 2019; Oudelaar et al., 2019). Studies often report limited characterization of the contents of PRP (Fice et al., 2019). Additionally, donor variability impacts not only the cell profiles of the final product, but also secretory components such as growth factors (Mazzocca et al., 2012).

In contrast, serum-derived products such as autologous conditioned serum (ACS) (Hraha et al., 2011; Evans et al., 2016) or hyperacute serum (hypACT) (Jeyakumar et al., 2017; Kuten et al., 2018) are generated by intentional extracorporeal clotting of whole blood via exposure to glass surfaces without the addition of an anticoagulant. Therefore, their profile of molecular components is more similar to the physiological process of hemostasis. In addition, serum products are considered to be cell-free (Wehling et al., 2017). Again, there are substantial differences in the production protocols with clotting times of up to a day for ACS or as short as 10 min for hypACT. As a result of clotting, the sera are deprived of coagulation factors, but enriched in cytokines, such as interleukin-1 (IL-1) receptor antagonist, and growth factors including TGF-β shed from intracellular reservoirs in mononuclear cells or platelets (Meijer et al., 2003; Ríos et al., 2015; Kardos et al., 2019). Similar to plasma derivatives, there are commercial devices available that support the preparation of serum derivatives from autologous whole blood (Baltzer et al., 2009; Kardos et al., 2018).

Besides growth factors, blood products contain extracellular vesicles (EVs) which are a heterogeneous group of nanoscale cell-derived membrane vesicles. On the one hand, they are generated inside multivesicular bodies (exosomes) and are shed upon fusion of these organelles with the plasma membrane. On the other hand, they can be formed from membrane blebs at the cell surface that bud off the plasma membrane (microvesicles). Once EVs have been released, it is almost impossible to discriminate between exosomes and microvesicles, because molecular markers that uniquely identify a given EV subtype are lacking up to date. Nevertheless, plasma membrane–derived vesicles often expose phosphatidylserine (Morel et al., 2011), whereas higher amounts of tetraspanins such as CD9, CD63, or CD81 are found in EVs originating from the cell interior (Andreu and Yáñez-Mó, 2014). EVs have been detected in all body fluids including blood, urine, breast milk, or synovial fluid (Qin and Xu, 2014; Boere et al., 2016).

Current research on regenerative approaches for cartilage recovery focuses on EVs derived from mesenchymal stem/stromal cells (MSCs) obtained from adipose tissue (A-MSC), bone marrow (BM-MSCs), or embryonic stem cells (ESC-MSCs) (D’Arrigo et al., 2019; Li et al., 2019). EVs from A-MSCs were shown to increase cartilage matrix synthesis indicated by type II collagen (COL2A1) expression and reduced expression of catabolic enzymes such as matrix metalloproteinase 13 (MMP13) and a disintegrin and metalloproteinase with thrombospondin motifs 5 (ADAMTS5) in OA chondrocytes (Wu et al., 2019), while reducing inflammation via promoting transition to the M2 phenotype of macrophages (Lo Sicco et al., 2017). BM-MSC EVs were shown to promote chondrogenic SRY-box transcription factor 9 (SOX9) expression while downregulating MMP13 expression in OA chondrocytes (Sun et al., 2019). Similarly to A-MSC EVs, BM-MSC EVs increase cartilage matrix deposition including aggrecan (ACAN) expression and decrease inflammation (Robbins and Morelli, 2014; Vonk et al., 2018). Finally, ESC-MSCs recapitulate these observations with inhibition of apoptosis, increased COL2A1 expression and decreased type I collagen (COL1A1), and ADAMTS5 expression in OA chondrocytes (Wang Y. et al., 2017; Zhang et al., 2018). These experiments highlight the fact that the regenerative potential of the MSC treatments is mediated by EVs or conditioned medium rather than by cells. However, there is limited knowledge available about the chondroprotective effects mediated by blood-derived EVs, especially with respect to mechanisms of action of autologous blood products, and this study is the first report on direct effects of EVs on OA chondrocytes to our knowledge.

Our aim was to characterize EVs from citrate-anticoagulated platelet-rich plasma (CPRP) or hypACT, outline their impact on chondrotypic gene expression and proinflammatory signaling. We highlight that different blood products contain different residual cell constituents and different cellular origin of EV populations. Chondroprotective and chondrogenic gene expression changes on mRNA and protein level as well as modulation of proinflammatory signaling elicited in patient-derived OA chondrocytes exposed to full blood products compared to enriched blood product EVs revealed that EVs are sufficient to induce substantial gene expression changes and can even outperform full blood products.

## Materials and Methods

### Preparation of Blood Products

Human whole blood was drawn in-house by approved personnel from voluntary donors after informed consent and approval by the local ethics committee (ESC1020/2020). Donor inclusion criteria were age between 25 and 45 years, as well as healthy based on a self-evaluation form asking for primary diseases such as diabetes or conditions including underweight or pregnancy at time of blood donation. Selection of donors based on gender was done randomly to avoid gender bias. Blood was drawn from 10 donors, 6 female and 4 male. For CPRP preparation, blood was collected from 5 donors per round into citrate-coated vacutainer tubes (VACUETTE^®^ 9NC trisodium citrate 3.2%, Greiner BioOne, #455322) and processed as described earlier (Kuten et al., 2018). For preparation of hypACT, blood was collected using the hypACT^TM^ inject device developed by OrthoSera GmbH (Krems, Austria) and spun immediately at 1,710 × *g* for 8 min at room temperature (RT). Following the formation of platelet-rich fibrin in the device, the fibrin clot was squeezed by pressing the piston into the syringe to yield hypACT. Blood cell profiles in freshly prepared blood products were determined by a Sysmex XN-1000 Sa-01 cell counter. Blood products were processed immediately.

### EV Enrichment via Ultracentrifugation

Freshly prepared blood products (2.5 mL) were put into 15-mL polypropylene tubes (#91015) and centrifuged at 2,500 × *g* for 15 min at RT for preclearing from cells and cell debris. Aliquots of the precleared supernatant (S2 fraction) were saved for protein analysis. Throughout the study, phosphate-buffered saline (PBS) was free from Ca^2+^ and Mg^2+^ and was filtered with 0.22 μm sterile filters (Sartorius, #16534) before use. Aliquots of 2.5 mL cleared plasma or serum were mixed 1:1 PBS in ultracentrifugation tubes (Beckman-Coulter, #355647). After centrifugation at 100,000 × *g* for 120 min at 4°C in a MLA-80 fixed angle rotor (k-factor 29; 45,000rpm), pellets (P100 fraction) were resuspended PBS. Aliquots of the supernatant after ultracentrifugation (S100 fraction) and the floating fat fraction (fat) were stored at -80°C after determination of whole protein concentration of aliquots lysed in RIPA buffer via BioRad DC assay according to the manufacturer’s recommendations. We have submitted all relevant data of our experiments to the EV-TRACK knowledge base (EV200024) (Van Deun et al., 2017).

### Nanoparticle Tracking Analysis

Size and concentration of particles were determined via NTA (ZetaView, Particlemtrix) in scatter mode as previously described (Gardiner et al., 2013; Mehdiani et al., 2015). In brief, samples were diluted as required with PBS. Preacquisition camera settings were kept constant at sensitivity 80 and shutter 100. Videos were acquired at 11 positions each with 30 frames per second for 2 s in one acquisition cycle. Video analysis was done with ZetaView 8.04.02 software and using postacquisition parameters of 20 minimum brightness; minimum and maximum area of 5 and 1,000, respectively; and 64 classes per decade.

### Flow Cytometry

Aliquots of freshly prepared blood products were directly used for staining. Blood products were diluted 100-fold in PBS and stained for 15 min with 1 μL lactadherin-FITC (Haematologic Technologies, #BLAC-FITC), 2 μL CD41-PC7 (Beckman-Coulter, #6607115), 2 μL CD235a-APC-AF750 (Beckman-Coulter, #A89314), and 5 μL CD14-PE (Beckman-Coulter, #A07764). After staining with fluorochrome, 400 μL PBS were added, and fluorescence in samples was measured by a Gallios cytometer. Calibration was performed with 1-, 0.5-, and 0.3-μm fluorescent-green silica particles at an excitation/emission wavelength of 485/510 nm (Kisker Biotech, Germany). Forward scatter was used as trigger signal. The EV gate was set below the 1-μm bead cloud as shown in Supplementary Figure S1 and previously described (Weiss et al., 2014; Fendl et al., 2018). Buffer, isotype, and single-stain controls are given in Supplementary Figure S1. The flow rate was set to 30 μL/min and acquisition time to 3 min. Data were analyzed using Kaluza software. To avoid carryover effects, a 2 min washing step with 0.1 μm sterile filtered double-distilled water was performed between each measurement at a flow rate of 60 μL/min. To confirm that the signals in the EV fraction were indeed dependent on the presence of intact EVs, a detergent lysis control was included by treatment of CPRP with 0.25% TritonX-100 to lyse vesicles, as shown in Supplementary Figure S1.

### Western Blot

Extracellular vesicle suspensions (10 μg total protein) were separated on 4–12% sodium dodecyl sulfate–polyacrylamide gel electrophoresis (Invitrogen, #NP0322) under reducing [for Alix, ApoB100, nuclear factor κB (NF-κB), COX2, SOX9, and GAPDH] or non-reducing (for CD9, CD63, and ApoA1) conditions via adding 100 mM dithiothreitol and probed for ApoA1 (Santa Cruz, #sc-376818), Alix (Cell Signaling, #2171), CD63 (BioLegend, #353005, clone H5C9), CD9 (SystemBiosciences, #EXOAB-CD9A-1), NF-κB (Santa Cruz, #sc-8008), COX2 (Cell Signaling, #12282T), SOX9 (Santa Cruz, #sc-20095), and GAPDH (Abcam, #ab37168), each 1:1,000 in 1% bovine serum albumin in PBST (PBS + 0.1% Tween 20). Proteins were detected via enhanced chemiluminscence (ECL) using WesternBright ECL substrate (Advansta, #K-12045-D20). Automatic white correction was applied to blot images via GIMP software version 2.8 before semiquantitative analysis was performed densitometrically using the GelAnalyzer plugin of ImageJ software version 1.51s.

### Chondrocyte Cell Culture

Osteoarthritis cartilage was obtained from four patients undergoing total knee replacement surgery after informed consent with approval by the local ethics committee (GS1-EK-4/480-2017). Chondrocytes were isolated and cultured as described previously (Bauer et al., 2016; De Luna-Preitschopf et al., 2017).

### EV Treatment of OA Chondrocytes

Isolated chondrocytes with maximum passage number of 2 were seeded into 6-well plates at a density of 1 × 10^4^ cells/cm^2^ in 3 mL growth medium (Dulbecco modified eagle medium) supplemented with 10% fetal calf serum (FCS), antibiotics, and ascorbic acid. After 24 h, medium was aspirated, cells rinsed with PBS, and culture was continued for 48 h in growth medium supplemented with either 10% pooled blood product (CPRP or hypACT), an equivalent amount of pooled isolated EVs from the respective blood product in serum-free conditions, or 10% FCS as control. Pooled blood products and EVs were used to limit donor variability. For hypACT EV treatment, media were supplemented with 1.42 × 10^9^ ± 2.12 × 10^6^ (SD) EVs. The amount of added CPRP EVs per well was 1.42 × 10^9^ ± 2.95 × 10^7^ (SD) EVs.

### Gene Expression Analysis via Reverse Transcriptase–Quantitative Polymerase Chain Reaction

EV-treated OA chondrocytes were washed with PBS once before RNA isolation using the High Pure RNA Isolation Kit (Roche, #11828665001). cDNA was synthesized via Transcriptor cDNA Synth Kit (Roche, #04897030001) according to the manufacturer’s protocols using 12 μL isolated RNA. For quantitative polymerase chain reaction (qPCR), FastStart Essential DNA Probes Master (Roche, #06402682001) was mixed with 1 μL cDNA and 900 nM of primers listed in Table 1. Ct values were determined on a LightCycler 96 instrument (Roche, #05815916001). Data were normalized to expression levels of GAPDH, and fold changes between conditions were calculated via the ΔΔCt method.

**TABLE 1:** Primer sequences and annealing temperatures (Ta) used in the study.

Gene	Forward	Reverse	T_a_ [°C]
COL1	gggattccctggacctaaag	ggaacacctcgctctccag	61
COL2	gtgtcagggccaggatgt	tcccagtgtcacagacacagat	60
MMP3	caaaacatatttctttgtagaggacaa	ttcagctatttgcttgggaaa	59
SOX9	tacccgcacttgcacaac	tctcgctctcgttcagaagtc	59
ACAN	cctccccttcacgtgtaaaa	gctccgcttctgtagtctgc	55
GAPDH	ctctgctcctcctgttcgac	acgaccaaatccgttgactc	62

### Enzyme-Linked Immunosorbent Assay

Concentrations of IL-6, tumor necrosis factor α (TNF-α), and IL-1β were determined in cell culture supernatants via ABTS-based enzyme-linked immunosorbent assay (Peprotech; #900-K16, #900-K25, and #900-K95). Cell culture supernatants were measured undiluted or diluted 1:50 with PBS. Assays were performed as recommended by the manufacturer. Absorbance was measured on a BioTek Synergy 2 plate reader, and cytokine concentrations were calculated via BioTek Gen5 software version 1.11.5.

### Statistical Analysis

Unless otherwise stated, data were tested with paired two-sided *t*-test using GraphPad version 8.4.2. Statistical significance was accepted for *p* < 0.05. Gene expression data of EV-treated chondrocytes were tested with multiple *t*-test accepting an false discovery rate of 10% (*q* < 0.1) determined using the two-step linear step-up procedure of Benjamini et al. (2006).

## Results

### Blood Cell Profiles of Blood Products

**FIGURE 1:**
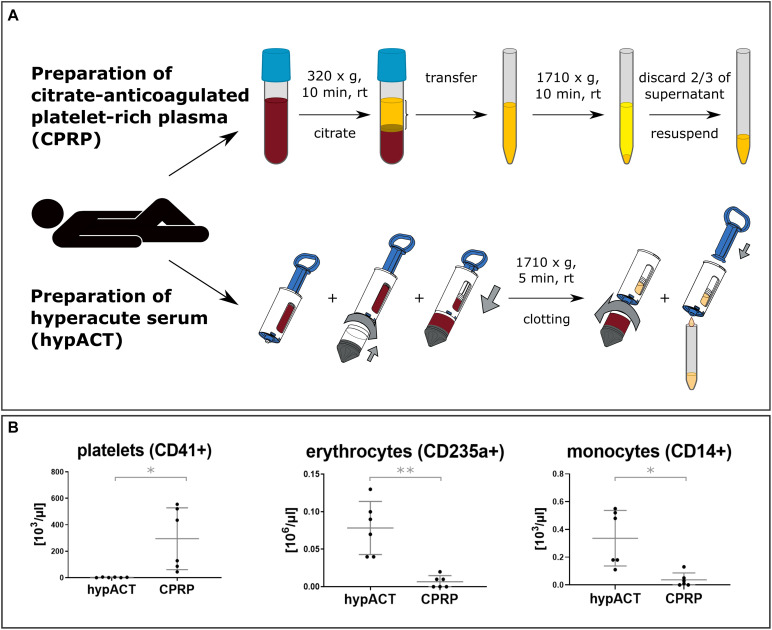
**(A)** Overview of employed blood product production procedures for citrate-anticoagulated platelet-rich plasma (CPRP) and hyperacute serum (hypACT). Two rounds of centrifugation are involved and anticoagulant (citrate) is added for CPRP, whereas hypACT does not require anticoagulation and only one centrifugation step. **(B)** Profiles of residual blood cells in blood products. Data from *n* = 6 donors are shown with SD error bars. *p < 0.05; **p < 0.01.

As outlined in Figure 1A, the procedures to obtain CPRP and hypACT from whole blood focused on different subsets of blood fractions. Whole blood was drawn from each donor to prepare both blood products each time. CPRP production enriched platelets by two rounds of centrifugation in presence of the anticoagulant citrate, whereas hypACT was generated in a closed system involving blood clotting and squeezing serum components from the resulting fibrin clot without an anticoagulant. To screen freshly prepared blood products for residual cell profiles, a routine hematologic analysis was performed (see section “Materials and Methods*”*). As shown in Figure 1B, CPRP contained high amounts of platelets [282,000 per μL; 44,000–555,000 95% confidence interval (CI)], but low numbers of erythrocytes (5,000 per μL; 0–12,500 95% CI) or monocytes (20 per μL; 0–70 95% CI). In contrast, hypACT was in essence devoid of platelets [*t*(5) = 3.043; *p* = 0.0287] while containing significantly more erythrocytes [*t*(5) = 5.998; *p* = 0.0018] and monocytes [*t*(5) = 3.816; *p* = 0.0124] than CPRP.

### EV Cell Origin in Different Blood Products

**FIGURE 2:**
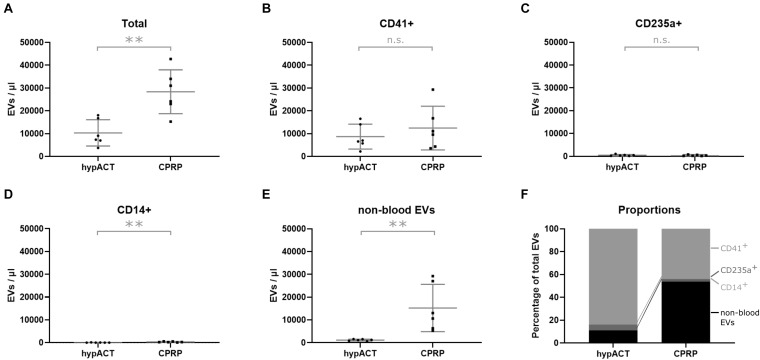
Tissue origin of EVs in blood products determined via flow cytometry. **(A)** Total extracellular vesicle (EV) concentration, **(B)** platelet-derived EVs (CD41^+^), **(C)** erythrocyte-derived EVs (CD235a^+^), **(D)** monocyte-derived EVs (CD14^+^), **(E)** non-blood EVs, **(F)** fractional composition of EV population by tissue origin. Data from n=6 donors are shown with SD error bars. **p < 0.01.

To characterize EVs in CPRP as well as in hypACT, their cellular origin was determined via flow cytometry using CD41 as platelet marker, CD235a as erythrocyte marker, and CD14 as monocyte marker. Lactadherin positivity (LA^+^) was used to differentiate EVs from background. LA^+^ events were subsequently analyzed for presence of cell surface markers indicating the cellular origin of the EVs (Figures 2A–E). The total EV concentration was significantly lower [*t*(5) = 3.255; *p* = 0.0226] in hypACT than in CPRP (Figure 2A). EVs were found to originate mainly from platelets in hypACT, whereas the majority of EVs in CPRP were not derived from blood cells (Figure 2B). However, the CD41^+^ EV concentrations were not significantly different between CPRP and hypACT [*t*(5) = 0.8929; *p* = 0.4128]. The contribution of erythrocyte-derived EVs (Figure 2C) or monocyte-derived EVs (Figure 2D) to the total EV concentrations was low. Nevertheless, there were significantly [*t*(5) = 4.793; *p* = 0.0049] more CD14^+^ EVs in CPRP (250 EVs/μL, 194–514 95% CI) than in hypACT (34 EVs/μL, 6–155 95% CI). CPRP contained a high concentration of CD41^–^/CD235a^–^/CD14^–^ EVs, which was significantly [*t*(5) = 3.251; *p* = 0.0227] lower in hypACT (Figure 2E). Around 84% of total EVs originated from platelets in hypACT, whereas platelet EVs constituted a 44% proportion of total EVs in CPRP (Figure 2F).

### Enrichment of EVs From Blood Products

**FIGURE 3:**
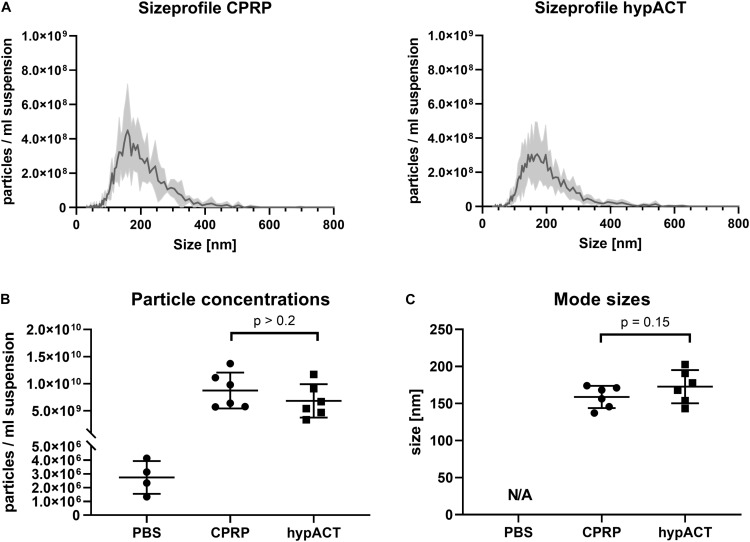
Characterisation of particles in pellets after ultracentrifugation (P100 fractions) via nanoparticle tracking analysis (NTA). **(A)** Size profiles of citrate-anticoagulated platelet-rich plasma (CPRP) and hyperacute serum (hypACT). **(B)** Total particle concentrations in the EV suspensions or eluent (PBS). **(C)** Mode size of particles determined in 6 Donors. Determination of mode size was not possible in PBS (N/A, not a number) as the number of recorded traces was too low, as expected, to reliably determine particle sizes. Data are given as mean ± SD.

EVs were enriched from CPRP and hypACT of 6 healthy donors. Following ultracentrifugation, particle size distributions were determined in EV suspensions to monitor the size and concentration of EVs from CPRP and hypACT via NTA (Figure 3A). The plots highlight that the concentration of EVs tended to be higher in CPRP than in hypACT after enrichment via ultracentrifugation, although not significantly [*t*(10) = 1.452; *p* = 0.2062] (Figure 3B). Similarly, average EV mode size was comparable between CPRP and hypACT [*t*(10) = 1.699; *p* = 0.15] (Figure 3C).

**FIGURE 4:**
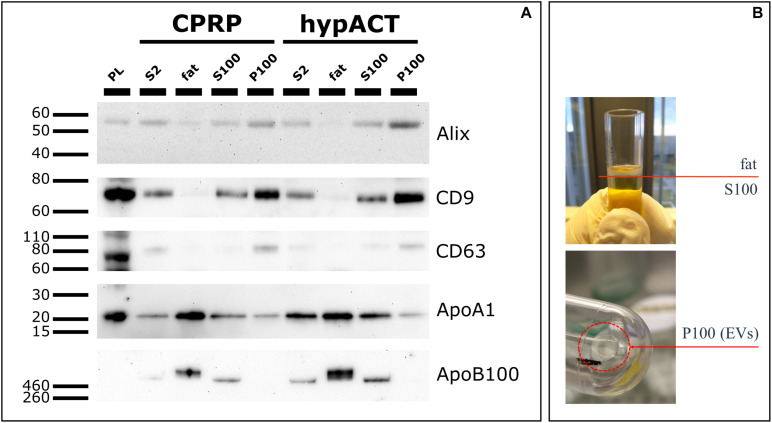
Characterisation of protein markers via Western Blot. **(A)** Extracellular vesicle (EV) markers (Alix, CD9, CD63) and lipoproteins (ApoA1, ApoB100) were detected in fractions obtained after ultracentrifugation (fat, S100, P100). Platelet lysate (PL) and S2 fraction (pre-cleared blood product supernatant) were used as positive controls. 10 μg total protein per lane, except for PL (20 μg) **(B)** Graphical representation of fractions fat, S100 and P100 obtained after an UC run.

Enriched EVs from CPRP and hypACT were probed for the EV markers Alix, CD9, and CD63, as well as the non-EV markers apolipoprotein A1 (ApoA1) and apolipoprotein B100 (ApoB100) on Western blot (Figure 4A) to show enrichment of EVs and depletion of non-EV factors as outlined in the MISEV 2018 guidelines (Thery et al., 2018). Fractions obtained from input material (S2) after ultracentrifugation are outlined in Figure 4B. Alix was enriched in P100 fractions while depleted in fat fractions compared to input control (S2) as expected. Densitometric analysis (Supplementary Figure S2) revealed a higher band intensity for Alix in hypACT P100 than CPRP P100 normalized to input controls (CPRP S2 or hypACT S2). Similarly, CD9 and CD63 were detected with higher intensity in P100 fractions compared to S2 for CPRP and hypACT, whereas fat fractions were in essence devoid of EV markers. In contrast, fat fractions were enriched for ApoA1 and ApoB100 lipoproteins. While P100 of both CPRP and hypACT was devoid of ApoB100, 15.7 or 19.7% of signal intensity of ApoA1 was obtained in P100 compared to the respective fat fraction in CPRP or hypACT. This indicates a partial depletion of high-density lipoprotein particles and depletion of low density lipoprotein (LDL) particles below the limit of detection from P100 fractions.

### EVs Elicit Chondroprotective Gene Expression and Reduce IL-6 Secretion

**FIGURE 5:**
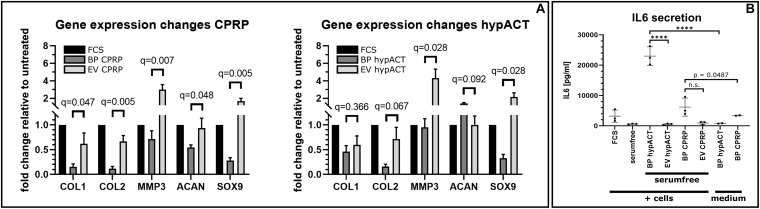
Gene expression changes mediated by citrate-anticoagulated platelet-rich plasma (CPRP) and hyperacute serum (hypACT) blood products (BP) and EVs in patient derived chondrocytes relative to untreated cells. Data are given as arithmetic mean of triplicate measurements ± SE of 4 individual chondrocyte donors.

To gain functional insights into EVs from blood products, OA patient-derived chondrocytes were treated with EVs enriched from CPRP and hypACT. Cell culture media were supplemented with full blood product or an equivalent amount of EVs in serum-free media, and gene expression changes of anabolic (COL2A1, SOX9, and ACAN), as well as catabolic markers (COL1A1, MMP3), were assessed via reverse transcriptase (RT)–qPCR (Figure 5A). Gene expression changes in chondrocytes in response to full blood product (BP CPRP) or CPRP EVs were normalized to untreated cells cultured in growth medium supplemented with 10% FCS, a standard supplement used in cell culture studies. Expression of all five tested markers was lower in CPRP blood product treated cells compared to FCS-treated cells. However, CPRP EVs significantly increased expression of all tested markers compared to CPRP blood product (BP CPRP) by a factor of 4.0 [*t*(6) = 2.049; *q* = 0.047], 5.6 [*t*(6) = 4.376; *q* = 0.005], 4.2 [*t*(6) = 3.700; *q* = 0.007], 1.7 [*t*(6) = 1.878; *q* = 0.048], or 6.0 [*t*(6) = 4.427; *q* = 0.005] on average for COL1A1, COL2A1, MMP3, ACAN, or SOX9, respectively. In contrast, hypACT EVs significantly promoted expression of COL2A1, MMP3, and SOX9 by around 4.5-fold [*t*(6) = 2.300; *q* = 0.067], 4.5-fold [*t*(6) = 3.271; *q* = 0.028], and 6.5-fold [*t*(6) = 3.690; *q* = 0.028] on average, respectively, compared to hypACT blood product (BP hypACT), while COL1A1 expression showed no significant change [*t*(6) = 0.6253, *q* = 0.366]. Interestingly, hypACT EVs decreased ACAN expression 0.7-fold [*t*(6) = 1.865; *q* = 0.092].

To observe effects of EVs on cytokine secretion, cell culture supernatants of unstimulated OA chondrocytes treated with full blood products or the respective EVs in serum-free medium were screened for IL-6, TNF-α, and IL-1β (Figure 5B). Analysis of variance and Tukey *post hoc* test revealed significant variation among IL-6 medium levels [*F*(7,14) = 51.96; *p* < 0.0001]. IL-6 concentration in EV-treated cell supernatants were not different to cells cultured in serum-free medium (*p* > 0.9999), supporting that EVs do not promote IL-6 secretion. Nevertheless, treatment of cells with full blood product elicited significant IL-6 secretion for hypACT (*p* < 0.0001), but not as strong for CPRP (*p* = 0.0487). IL-6 levels were determined in medium supplemented with either blood product to check whether the determined IL-6 was already present in the blood product or was secreted from cells. IL-6 predominantly originates from cells exposed to hypACT blood product (*p* < 0.0001), as 22,994 ± 3,083 (SD) pg/mL was found in the treatment group (BP hypACT), compared to 820.5 ± 117.8 (SD) pg/mL in the medium control. In contrast, 6,176 ± 2,997 (SD) pg/mL was determined in CPRP blood product–treated cell supernatant; however, IL-6 levels in CPRP medium control [3,422 ± 144.6 (SD) pg/mL] were not significantly different (*p* < 0.7062). TNF-α and IL-1β levels remained below limit of detection (data not shown).

### EV-Mediated Inhibition of Inflammation

**FIGURE 6:**
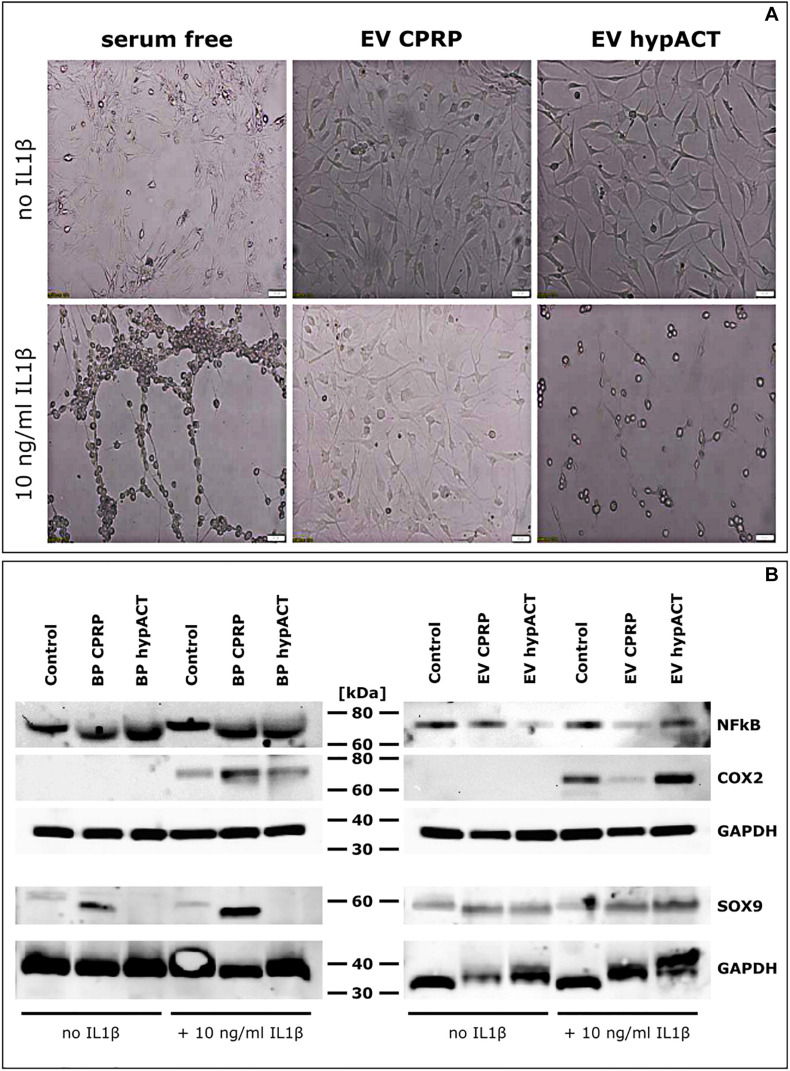
Impact of EVs on pro-inflammatory signaling in OA chondrocytes. **(A)** Morphological changes mediated by EVs from CPRP or hypACT in absence or presence of IL1β. Scale bars 100 μm. **(B)** Protein expression of NF-κB and COX2 in chondrocytes treated with blood products or EVs in absence or presence of IL1β compared to untreated cells assessed via Western Blot. **(C)** Quantification of interleukin 6 (IL6) in cell culture supernatants after 48 h via ELISA. Statistical significance was determined via one-way ANOVA and Tukey’s post hoc test with α = 0.05. Data are given as arithmetic mean ± SD of duplicate measurements of three independent chondrocyte donors. Medium controls were mesured in duplicate, both values are given, as the same medium was used for each donor. BP, blood product; ****p < 0.0001; n.s, not significant.

In parallel to gene expression analysis, impact of IL-1β–mediated proinflammatory signaling on chondrocytic gene expression in chondrocytes treated with full blood products or EVs from CPRP or hypACT was investigated in absence or presence of IL-1β in serum-free medium (Figure 6). Cell morphology of cells unstimulated with IL-1β was ameliorated by EVs of either CPRP or hypACT to a more healthy appearance in comparison to cells in serum-free medium. Chondrocytes showed notable morphologic changes in presence of IL-1β; however, the induced phenotype was prevented by the presence of CPRP EVs, but not hypACT EVs (Figure 6A). Protein expression of NF-κB, COX2 as well as SOX9 was monitored via Western blot (Figure 6B). NF-κB levels of control cells in serum-free medium were comparable to cells treated with full blood products with or without supplementation with IL-1β. Interestingly, NF-κB levels decreased in presence of hypACT EVs in absence of IL-1β, whereas NF-κB decreased in presence of EVs from both blood products in presence of IL-1β. Stimulation with IL-1β induced COX2 expression as expected. Stimulated cells treated with CPRP blood product increased COX2 expression. Nevertheless, CPRP EVs showed a stronger capacity to inhibit COX2 expression than hypACT EVs, which increased COX2 expression. While chondrocytes exposed to hypACT blood product did not express detectable levels of SOX9, hypACT EVs induced SOX9 expression. CPRP EVs decreased SOX9 expression compared to CPRP blood product in the presence of IL-1β. Expression of NF-κB, COX2, and SOX9 relative to GAPDH was determined via densitometry (Supplementary Figure S3), highlighting that SOX9 expression was strongest in IL-1β–stimulated cells in presence of CPRP blood product.

## Discussion

Because of their promising regenerative features, blood-derived products, especially PRP, are often applied to treat cartilage injuries and OA. Upon injection of PRP, a multitude of growth factors stored within the α-granules of platelets are released stimulating healing of the tissue. Until now, blood-derived products are mainly characterized based on their growth factor content, and their underlying mode of action is not clear yet. In addition, other active components including EVs, which also play a major role in mediating regenerative stimuli, are poorly investigated. The discovery of EVs in body fluids including blood and blood products has sparked interest of investigating EVs in regenerative medicine (Tao et al., 2017a). We investigated the cellular components and the cell origin of EVs of a plasma- and a serum-based blood product (Figure 1A). While CPRP expectedly contained large numbers of platelets and low amounts of erythrocytes or monocytes, we found basically no platelets in hypACT, while it contained significantly higher numbers of erythrocytes and monocytes than CPRP (Figure 1B). As erythrocytes and monocytes were pelleted in the first centrifugation step during CPRP production, they were not found in the PRP fraction. On the other hand, coagulation trapped platelets in the clot in hypACT; therefore, no platelets were found in the final blood product. However, we found low amounts of residual erythrocytes and monocytes, which escaped becoming trapped in the clot. This might be due to low fibrinogen or fibrin content below the boundary layer between pelleted cells and the clot in the hypACT^TM^ inject device. Therefore, a sparse fibrin mesh forms around cells that might still be present in the upper part of the device. Upon hypACT ejection, these weakly trapped cells might be washed out and give rise to the observed erythrocyte and monocyte content.

As observed via flow cytometry, the majority of EVs in hypACT was derived from platelets, whereas less than 50% of all EVs originated from platelets in CPRP, in contrast to previous reports (Brisson et al., 2017). Nevertheless, we found a significantly higher total EV concentration in CPRP than in hypACT (Figure 2A). As we defined EVs as lactadherin positive events in flow cytometry, all EVs included in our analysis exposed phosphatidylserine, which was reported to support thrombin formation and coagulation (Tripisciano et al., 2017). Therefore, we expected that hypACT contained lower amounts of platelet-derived EVs (CD41^+^) because of assisting in clot formation (Figure 2B). Presence of CD41^+^ EVs could be explained by shear–stress–induced release of EVs from platelets within the clot (Holme et al., 1997; Reininger et al., 2006). EVs derived from erythrocytes (CD235a^+^) or monocytes (CD14^+^) contributed only with 0.5–5% to the total EV content. Surprisingly, CPRP contained a large population of EVs for which we were not able to assign a blood cell origin, although they were lactadherin-positive (LA^+^), whereas EVs of non–blood cell origin represented less than 10% in hypACT (Figure 2E). We propose that LA^+^ EVs assisted in clot formation and were trapped in the fibrin mesh similar to platelets. However, this would likely not change the proportional composition of EV populations, as the platelet-derived EVs would be trapped as well. An explanation for the proportional change could be a release of EVs from platelets after clotting as discussed above. Therefore, the clotting process might be responsible for the observed changes of EV populations between plasma and serum (Figure 2F).

Plasma and serum are complex mixtures, and until now, it is not clear which components are responsible for their regenerative capacity. We applied ultracentrifugation to obtain small and large EVs devoid of other blood product components including lipoproteins. The particle concentration in P100 pellets of CPRP and hypACT turned out to be non-significantly different according to NTA (Figure 3), while it was found significantly higher in CPRP than hypACT via flow cytometry before isolation (Figure 2A). Ultracentrifugation leads to aggregate formation (Linares et al., 2015), and CPRP EVs might be differently affected by this effect than hypACT EVs. Although aggregate formation would result in an increase of particle mode size, we found no significant difference (Figure 3C). However, the preparation procedure resulted in enrichment of EV marker proteins as shown on Western blot (Figure 4). While EV markers were enriched in the P100 fraction as anticipated, lipoproteins were floating in a buoyant fat layer on top. This fraction was expected to be enriched for ApoB100, the apolipoprotein of LDL; however, an enrichment of ApoA1 was observed as well. This indicates that our ultracentrifugation protocol results in pure EV fraction depleted of lipoproteins.

Although the effects of EVs derived from mesenchymal stem cells on cartilage regeneration have been investigated *in vitro* (Vonk et al., 2018; Kim et al., 2019), and *in vivo* (Cosenza et al., 2017), the regenerative potential of autologous blood product–derived EVs has not been studied yet. Treatment of patient-derived OA chondrocytes with CPRP EVs promoted expression of chondrogenic markers COL2A1, ACAN, and SOX9, but also elevated COL1A1 and MMP3 expression, which stimulates or maintains loss of function of hyaline cartilage in OA (Miosge et al., 2004; Eldridge et al., 2016). MMP3 rather than MMP13, which is considered as hallmark gene for OA (Wang et al., 2013), was chosen as target as MMP3 is more prominently expressed in OA compared to MMP13 (Bau et al., 2002); can activate other MMPs including MMP13 (Murphy et al., 1999); and might be more sensitive to immediate gene expression changes. Increased COL1A1 expression favors the formation of fibrocartilage leading to loss of function of articular cartilage, whereas MMP3 cleaves extracellular matrix (ECM) components such as various types of collagens, elastin, and proteoglycan core proteins including ACAN (Wu et al., 1991; Ravanti and Kahari, 2000; Troeberg and Nagase, 2012). In addition, it activates MMP13, the hallmark gene in OA (Wang et al., 2013), which even more drives cartilage degradation. Other targets are cytokines such as TNF-α and IL-1β, which are both involved in OA progression (van de Loo et al., 1995; Wehling et al., 2009; Kapoor et al., 2011). However, MMP3 might slow down articular cartilage destruction and inflammation as the mentioned cytokines synergize in the induction of MMP3 (Kelley et al., 2007). Therefore, temporarily increasing MMP3 expression via EVs forms a negative feedback loop integrating catabolic tissue remodeling with anti-inflammatory effects. In contrast, anabolic gene expression was driven by CPRP EVs as well. SOX9 was strongly increased, which promotes chondrocyte differentiation, cartilage formation, and induction of COL2A1 (Bi et al., 1999) shifting cartilage tissue homeostasis toward a more physiologic phenotype. Interestingly, OA chondrocytes responded with increased COL2A1, MMP3 and SOX9 expression to hypACT EVs compared to full blood product as well. Similar to CPRP EVs, MMP3 and SOX9 were strongly upregulated by hypACT EVs. However, hypACT blood product was more effective at inducing ACAN expression than hypACT EVs. Nevertheless, hypACT EVs did not increase COL1A1 expression in contrast to CPRP EVs compared to the respective blood product. Therefore, a more favorable disease modifying effect for cartilage homeostasis is expected for hypACT EVs as the COL1A1-based fibrocartilage aspect might be lower. Interestingly, an increase of MMP3 expression was also found in response to EVs from adipose-derived MSCs, which was considered beneficial for regulating ECM remodeling and tissue reconstruction (Wang L. et al., 2017).

The gene expression analysis used cells cultured in growth medium supplemented with 10% FCS as untreated reference condition. It is clear that exposure to FCS affects chondrocytes differently than presence of PRP (Jeyakumar et al., 2017), and FCS is not a clinically applicable treatment option; however, it was used in the study to highlight differences of gene expression between blood product and the respective EV preparation.

Presence of EVs altered the morphologic appearance of cultured cells. While cells unstimulated by IL-1β responded to EVs of either CPRP or hypACT with a morphologic change to a more healthy appearance (Figure 6A), stimulation with IL-1β induced a morphological phenotype indicative of cell death, which was rescued by CPRP EVs, but not hypACT EVs. This observation coincides with COX2 protein expression changes in response to IL-1β, where CPRP EVs clearly decreased COX2 expression, whereas hypACT EVs unexpectedly increased COX2 expression (Figure 6B). On the other hand, CPRP blood product increased COX2 protein expression. However, another study suggested decreased COX2 expression, although on mRNA level, in response to CPRP blood product during a 48-h observation period as in our study (Moussa et al., 2017). Interestingly, only CPRP blood product induced SOX9 protein expression in contrast to hypACT blood product. Nevertheless, SOX9 was markedly induced by hypACT EVs, whereas CPRP EVs decreased SOX9 expression compared to CPRP blood product. As IL-1β inhibits SOX9 expression (Murakami et al., 2000) and hypACT EVs were able to counteract this effect, one can conclude that hypACT EVs have a strong chondrogenic potential. This observation inversely correlated with increased IL-6 secretion in presence of hypACT blood product compared to hypACT EVs (Figure 5B), which totally prevented IL-6 secretion. However, this finding contrasts a previous work that showed increased SOX9 expression and chondrogenic differentiation of MSCs mediated by increased IL-6 signaling (Kondo et al., 2015). Nevertheless, elevated IL-6 signaling is found in OA (Akeson and Malemud, 2017). Therefore, preventing IL-6 secretion while increasing SOX9-mediated chondrogenic effects in OA chondrocytes by hypACT EVs is highly favorable.

Limitations of this study include a short treatment period before analysis. To investigate time-resolved effects of EVs, sampling over a longer treatment period or repeated treatments would provide more detailed evidence of EV treatment outcomes in the long run. However, the short treatment strategy is a consequence of the second limitation of the *in vitro* model, as primary chondrocytes were early found to adapt a fibroblastic phenotype on flat plastic surfaces during extended periods of time (Holtzer et al., 1960; Green, 1971). Supplementation of growth media with FCS is common practice in cell culture studies involving chondrocytes, although it reduces SOX9 expression over time (Malpeli et al., 2004). Future studies involving long-term treatment of chondrocytes with EVs might substitute FCS-containing media with serum-free media (Barbero et al., 2003). Our *in vitro* model focuses on chondrocytes; however, several cell types are present in the joint, which come in contact with blood products or EVs upon intra-articular injection and impact the inflammatory state during disease. Nevertheless, they are the main target in cartilage regeneration as they produce ECM.

In summary, these results suggest that CPRP blood product should be applied in OA to achieve chondrogenesis rather than hypACT blood product, if elevation of inflammation driven by CPRP can be accepted. CPRP EVs might provide beneficial anti-inflammatory effects in acute OA, whereas hypACT EVs might be more suitable to drive chondrogenesis and cartilage regeneration in chronic OA. Although hypACT EVs and blood product promote inflammation indicated by COX2 expression and IL-6 secretion, respectively, this might be beneficial. As low-grade inflammation is characteristic to OA (Robinson et al., 2016) and might trap cartilage regeneration in a self-perpetuating loop, a short-term inflammatory boost might break the vicious circle to allow progression to later stages of wound healing, dampening inflammation and promoting tissue homeostasis, similar to the effects of the platelet secretome including EVs in wounds after blood coagulation and platelet lysate (Golebiewska and Poole, 2015; Nguyen et al., 2018; Carluccio et al., 2020).

Isolated EVs from various sources have been suggested as therapeutic agents in the context of cartilage recovery including EVs from human embryonic MSCs for osteochondral regeneration (Zhang et al., 2016) or MSC-derived EVs in OA (Toh et al., 2017). However, autologous EVs from blood products offer benefits over MSC EVs as there is no requirement for cell culture, no involvement of cell therapy and possibly associated adverse effects due to uncontrolled differentiation of MSCs such as undesired ossification (Savkovic et al., 2014), or no risk of transmission of diseases from donor to recipient. EV administration can be standardized in terms of concentration of EVs compared to full blood products. Reports on EV-based therapies in OA therapy are limited to MSC EVs so far (Li et al., 2018); however, blood-derived EVs are considered an emerging therapeutic approach in musculoskeletal conditions, tissue repair, and regeneration (Tao et al., 2017b; Murphy et al., 2018), and our work pioneers into the field of chondroregeneration using autologous EVs from blood.

## Data Availability Statement

All datasets generated for this study are included in the article/Supplementary Material.

## Ethics Statement

The studies involving human participants were reviewed and approved by the Lower Austrian Ethics Committee and the Ethics Committee of the Danube University Krems. The patients/participants provided their written informed consent to participate in this study.

## Author Contributions

AO developed the EV isolation protocol, performed the EV isolations, and did the NTA assessments and Western blot. KK performed the RT-PCR. CS drew the blood. OK-P prepared the blood products. RW performed the flow cytometry. AO and AD prepared the manuscript, while CS, ZL, VW, and SN provided valuable ideas, carefully read, and approved the manuscript draft. All the authors contributed to the article and approved the submitted version.

## Conflict of Interest

ZL is CEO and OK-P is employee of OrthoSera GmbH developing the hypACT^TM^ inject device. The remaining authors declare that the research was conducted in the absence of any commercial or financial relationships that could be construed as a potential conflict of interest.

## Supplementary Material

Supplementary Figure 1 Extracellular vesicle (EV) gating strategy and control experiments for flow cytometric characterisation of EVs in native blood products. **(A)** The EV gate was set around the 1 μm silica bead cloud as described in the Methods section. Forward scatter vs. side scatter (FS vs. SS) dot plots of silica beads (left) and of citrate-anticoagulated platelet-rich plasma (CPRP) (middle) as well as a lactadherin vs. CD41 density plot of CPRP (right) are shown. **(B)** Blood products were stained with FITC-conjugated lactadherin, (PC7)-conjugated anti-CD41 monoclonal antibody (mAb) as platelet marker, (APC-AF750)-conjugated anti-CD235a monoclonal antibody as erythrocyte marker and (PE)-conjugated anti-CD14 monoclonal antibody as monocyte marker were used to monitor cell origin of EVs. Buffer, fluorochrome-labeled reagent controls and single stainings of CPRP are shown. Bars indicate positive expression. **(C)** CPRP was treated with 0.25% TritonX-100 during staining as detergent lysis control, abolishing all signals in the EV gate and confirming the presence of vesicles.

Supplementary Figure 2 Densitometric analysis of indicated EV marker proteins and lipoproteins from Fig 4A. Data are given as proportional band intensity relative to the respective blood product after pre-clearing (CPRP S2 fraction or hypACT S2 fraction).

Supplementary Figure 3 Densitometric analysis of NFκB, COX2 and SOX9 expression relative to GAPDH in IL1β stimulated and unstimulated cells from Figure 6B. Data are given as ratio of the band intensity of the indicated marker relative to GAPDH band intensity. SOX9 intensity was normalised to the lower GAPDH bands.
