# Supplementary figures and images for: Characterization and Chondroprotective Effects of Extracellular Vesicles From Plasma- and Serum-Based Autologous Blood-Derived Products for Osteoarthritis Therapy
Source: Front Bioeng Biotechnol. 2020 Sep 25;8:584050. doi: 10.3389/fbioe.2020.584050 (PMC7546339; doi:10.3389/fbioe.2020.584050)

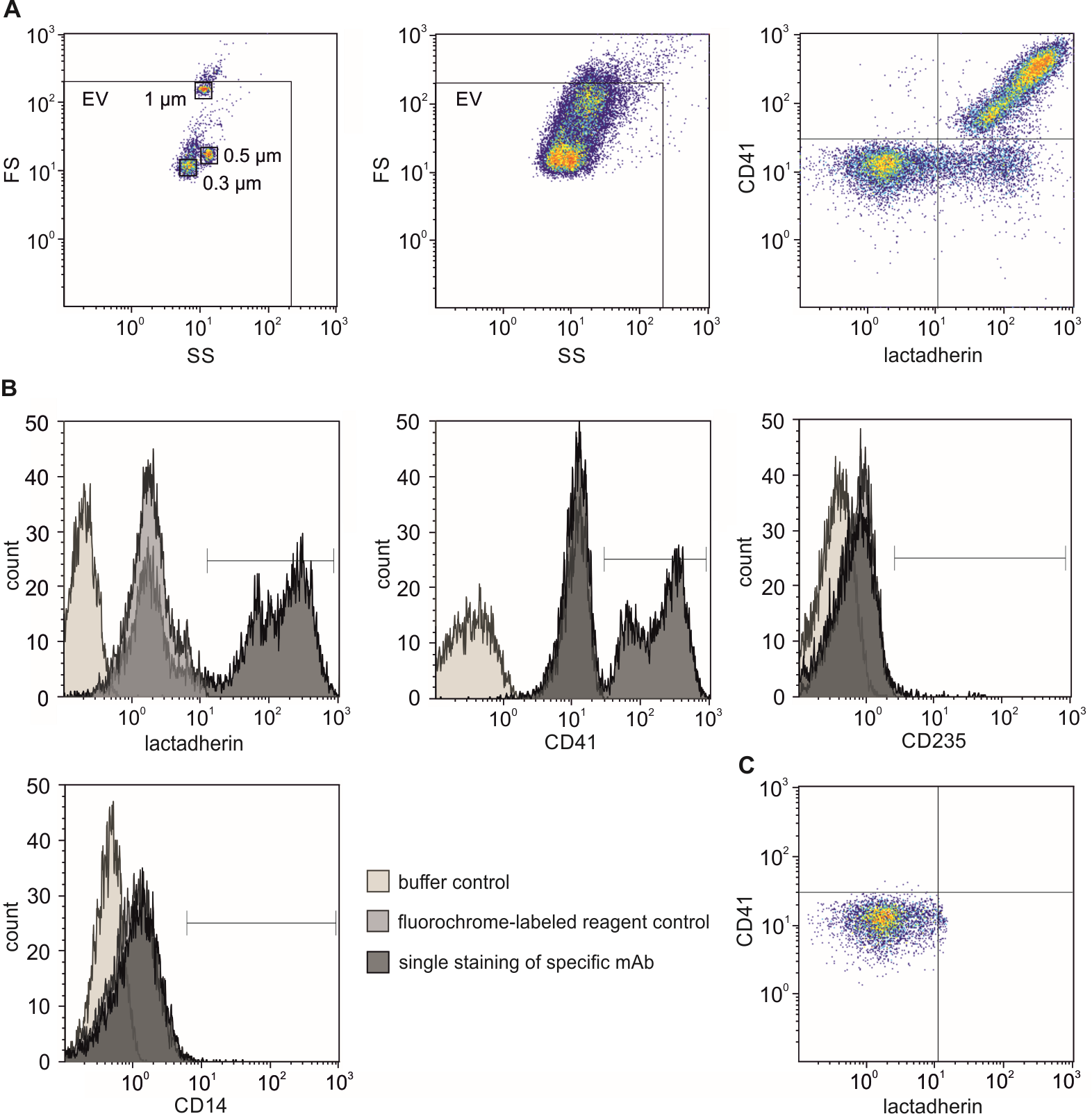

Supplement: Supplementary Figure 1 — Extracellular vesicle (EV) gating strategy and control experiments for flow cytometric characterisation of EVs in native blood products. (A) The EV gate was set around the 1 μm silica bead cloud as described in the Methods section. Forward scatter vs. side scatter (FS vs. SS) dot plots of silica beads (left) and of citrate-anticoagulated platelet-rich plasma (CPRP) (middle) as well as a lactadherin vs. CD41 density plot of CPRP (right) are shown. (B) Blood products were stained with FITC-conjugated lactadherin, (PC7)-conjugated anti-CD41 monoclonal antibody (mAb) as platelet marker, (APC-AF750)-conjugated anti-CD235a monoclonal antibody as erythrocyte marker and (PE)-conjugated anti-CD14 monoclonal antibody as monocyte marker were used to monitor cell origin of EVs. Buffer, fluorochrome-labeled reagent controls and single stainings of CPRP are shown. Bars indicate positive expression. (C) CPRP was treated with 0.25% TritonX-100 during staining as detergent lysis control, abolishing all signals in the EV gate and confirming the presence of vesicles. [file Image_1.TIF]

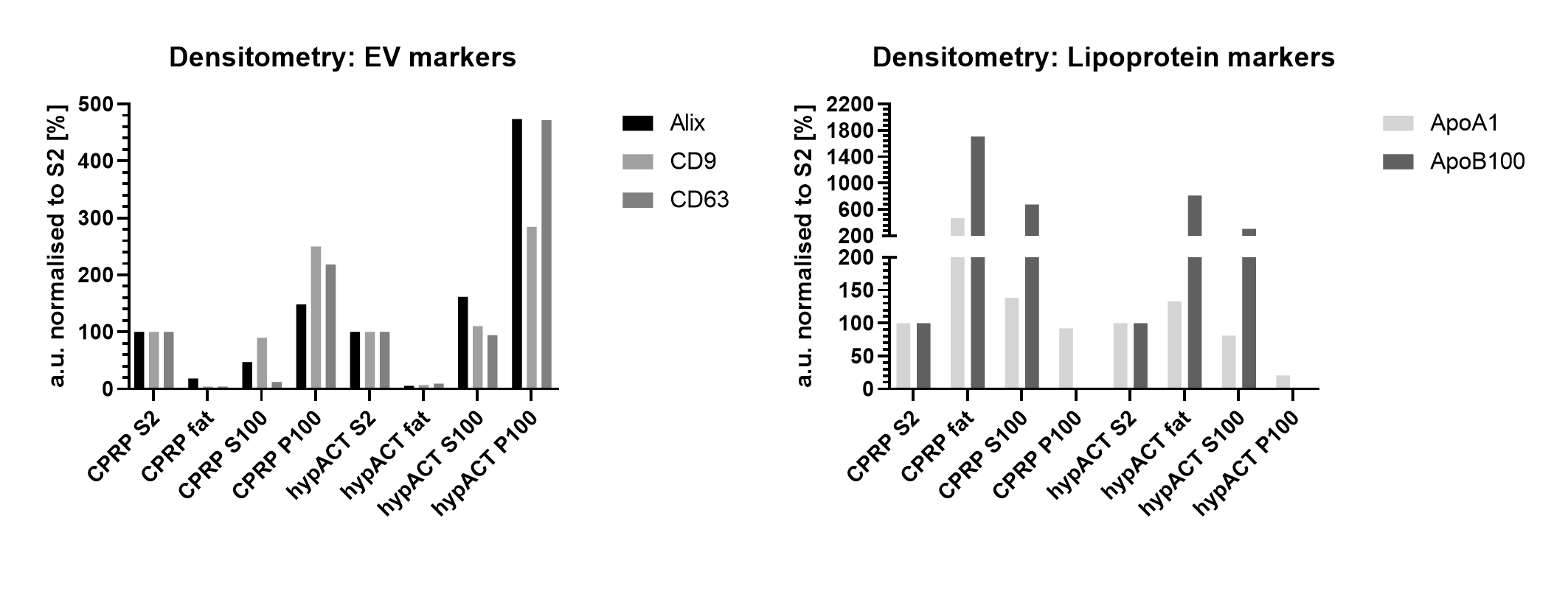

Supplement: Supplementary Figure 2 — Densitometric analysis of indicated EV marker proteins and lipoproteins from Fig 4A. Data are given as proportional band intensity relative to the respective blood product after pre-clearing (CPRP S2 fraction or hypACT S2 fraction). [file Image_2.TIF]

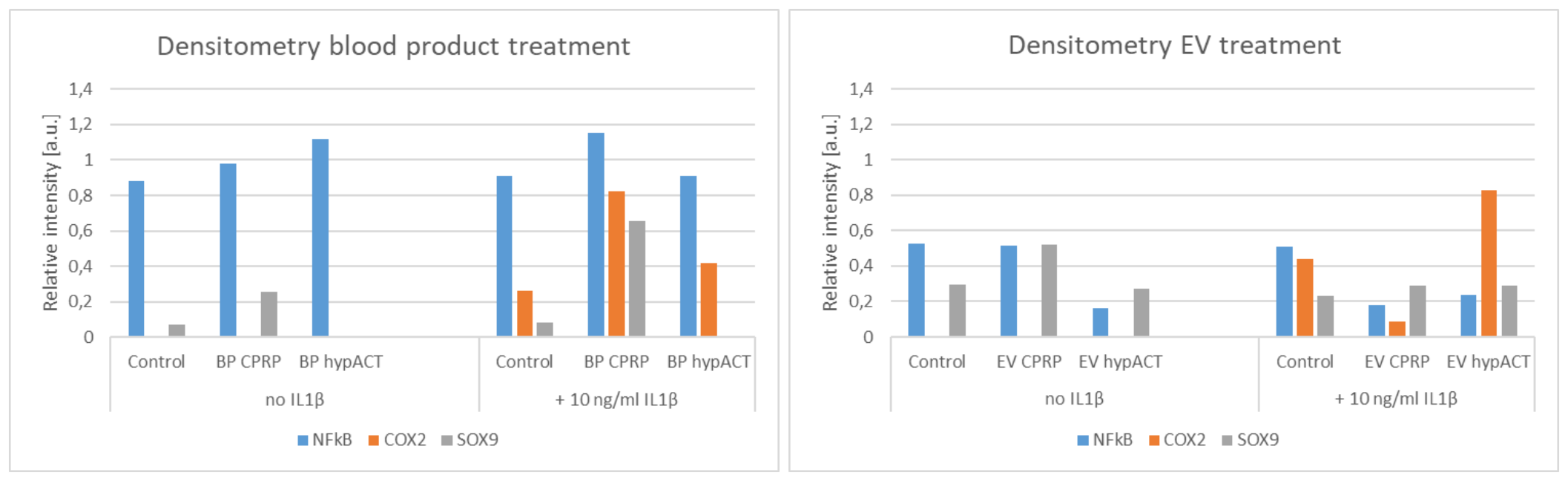

Supplement: Supplementary Figure 3 — Densitometric analysis of NFκB, COX2 and SOX9 expression relative to GAPDH in IL1β stimulated and unstimulated cells from Figure 6B. Data are given as ratio of the band intensity of the indicated marker relative to GAPDH band intensity. SOX9 intensity was normalised to the lower GAPDH bands. [file Image_3.TIF]
